# A Novel Homozygous Missense Mutation in the Zinc Finger DNA Binding Domain of GLI1 Causes Recessive Post-Axial Polydactyly

**DOI:** 10.3389/fgene.2021.746949

**Published:** 2021-10-15

**Authors:** Muhammad Umair, Farooq Ahmad, Saeed Ahmad, Qamre Alam, Mohd Rehan, Amany I. Alqosaibi, Mashael M. Alnamshan, Misbahuddin M Rafeeq, Shahnaz Haque, Ziaullah M Sain, Muhammad Ismail, Majid Alfadhel

**Affiliations:** ^1^ Medical Genomics Research Department, King Abdullah International Medical Research Center (KAIMRC), King Saud bin Abdulaziz University for Health Science, Ministry of National Guard-Health Affairs (MNGHA), Riyadh, Saudi Arabia; ^2^ Department of Chemistry, Women University Swabi, Swabi, Pakistan; ^3^ Consultant Orthopedic Surgeon, Capital Hospital Islamabad, Islamabad, Pakistan; ^4^ King Fahd Medical Research Center, King Abdulaziz University, Jeddah, Saudi Arabia; ^5^ Department of Medical Laboratory Technology, Faculty of Applied Medical Sciences, King Abdulaziz University, Jeddah, Saudi Arabia; ^6^ Department of Biology, College of Science, Imam Abdulrahman Bin Faisal University, Dammam, Saudi Arabia; ^7^ Department of Pharmacology, Faculty of Medicine, Rabigh, King Abduaziz University, Jeddah, Saudi Arabia; ^8^ Department of Microbiology, Faculty of Medicine, Rabigh, King Abduaziz University, Jeddah, Saudi Arabia; ^9^ Genetics and Precision Medicine department (GPM), King Abdullah Specialized Children’s Hospital (KASCH), King Abdullah International Medical Research Center, King Saud Bin Abdulaziz University for Health Sciences (KSAU-HS), King Abdulaziz Medical City, Ministry of National Guard Health Affairs (MNG-HA), Riyadh, Saudi Arabia

**Keywords:** post-axial polydactyly type A, GLI1, whole exome sequencing, novel missense variant, PAPA8

## Abstract

**Background:** Polydactyly is a prevalent digit abnormality characterized by having extra digits/toes. Mutations in eleven known genes have been associated to cause nonsyndromic polydactyly: *GLI3, GLI1, ZRS regulating LMBR1, IQCE, ZNF141, PITX1, MIPOL1, FAM92A, STKLD1, KIAA0825, and DACH1*.

**Method:** A single affected family member (IV-4) was subjected to whole-exome sequencing (WES) to identify the causal gene. Bi-directional Sanger sequencing was performed to segregate the identified variant within the family. *In silico* analysis was performed to investigate the effect of the variant on DNA binding properties.

**Results:** whole-exome sequencing identified a bi-allelic missense variant (c.1010C > T; p. Ser337Leu) in exon nine of GLI1 gene located on chromosome 12q13.3. With the use of Sanger sequencing, the identified variant segregated perfectly with the disease phenotype. Furthermore, in silico analysis of this DNA binding protein revealed that the variant weakened the DNA binding interaction, resulting in indecorous GLI1 function.

**Conclusion:** Herein, we report a novel variant in GLI1 gene, causing autosomal recessive post-axial polydactyly type A (PAPA) type 8. This confirms the critical role of GLI1 in digit development and might help in genotype–phenotype correlation in the future.

## Introduction

Polydactyly is also known as hexadactyly or polydactylism, which occurs due to extra complete on incomplete fingers or toes. It is one of the most common limb deformities observed prenatally or instantly after birth. The incidence of polydactyly is approximately 0.3–3.6/1,000 observed in live births and 1.6–10.7/1,000 in the general population (Akarsu et al., 1997; Umair et al., 2018). In addition, it has been observed that the frequency of involvement of the right hand is more than that of the left hand. Interestingly, numerous studies have reported that males are frequently (almost double) affected than females (Al-Qattan, 2012). Furthermore, the upper limbs are more affected than the lower, and the left foot is more affected than the right (Al-Qattan, 2012; Umair et al., 2018).

Broadly polydactyly is classified as a syndromic form that is associated with other phenotypes such as Bardet–Biedl syndrome (BBS), Ellis–Van Creveld (EVC) syndrome, split hand/foot malformation (SHFM), and syndactyly, while in nonsyndromic polydactyly, it is inherited as an isolated entity. Until now, more than 300 syndromic forms of polydactyly have been characterized (Hayat et al., 2020).

Moreover, polydactyly is further classified into three main types according to the severity of the duplication defect and position, viz., pre-axial polydactyly (PPD), where the additional digit is attached to the thumb or the big toe, or post-axial polydactyly (PAP), where the additional digit is located on the fifth finger. Furthermore, PAP is subclassified into post-axial type A with a fully developed digit and post-axial type B, where the extra digit is developed as a skin tag or rudimentary. Both type A and type B polydactyly may differ in penetrance, severity, and inheritance pattern (Umair et al., 2018, 2019, 2021; Hayat et al., 2020). The third and rare form of polydactyly is the complex polydactyly (mesoaxial polydactyly), where the extra digit originates between the second and fourth digits.

Several researchers also reported the incidence of PPD to be as high as 0.08 to 1.4 per 1,000 live births, while the prevalence of PAP is 1–2/1,000 live births with some ethnic group differences (Manske et al., 2017; Umair et al., 2019; Ullah et al., 2019a; Ullah et al., 2019b). In humans, until now, 11 genes (*GLI3*, *GLI1*, *ZRS* regulating *LMBR1*, *IQCE*, *ZNF141*, *PITX1*, *MIPOL1*, *FAM92A*, *STKLD1*, *KIAA0825*, and *DACH1*) and three other unknown loci have been mapped on different chromosome 13q21–32, 13q13.3–21.2, and 19p13.1–13.2, which have been linked with isolated polydactyly (Yousaf et al., 2020). However, eight of the 11 genes have been associated with isolated PAP type A including GLI family zinc finger three gene (*GLI3*; OMIM 174200; 7p14.1), zinc finger protein 141 gene (*ZNF141*; OMIM 615226; 4p16), GLI family zinc finger 1 (*GLI1*; OMIM 618123; 12q13.3), IQ Motif Containing E (*IQCE*; OMIM 617642; 7p22.3), KIAA0825 (OMIM 618498; 5q15), family with sequence similarity 92 member A1 (*FAM92A*; OMIM 618219; 8q22.1), and Dachshund Homolog 1 (*DACH1*; OMIM 603803) (Kalsoom et al., 2013; Umair et al., 2017, 2021) (Table 1).

**TABLE 1:** List of genes reported for isolated recessive PAPA until now.

Phenotype	Inheritance	Gene/locus	Location	MIM number	Gene/locus MIM number
PAPA6	AR	ZNF141	4p16.3	615226	194648
PAPA7	AR	IQCE	7p22.3	617642	617631
PAPA8	AR	*GLI1*	12q13.3	618123	165220
PAPA9	AR	FAM92A	8q22.1	618219	617273
PAPA10	AR	KIAA0825	5q15	618498	617266
PAPA11	AR	*DACH1*	13q21.33	603,803	603,803
PAPA1 and B	AD	*GLI3*	7p14.1	174200	165240

Note. PAPA, post-axial polydactyly type A.

**FIGURE 1:**
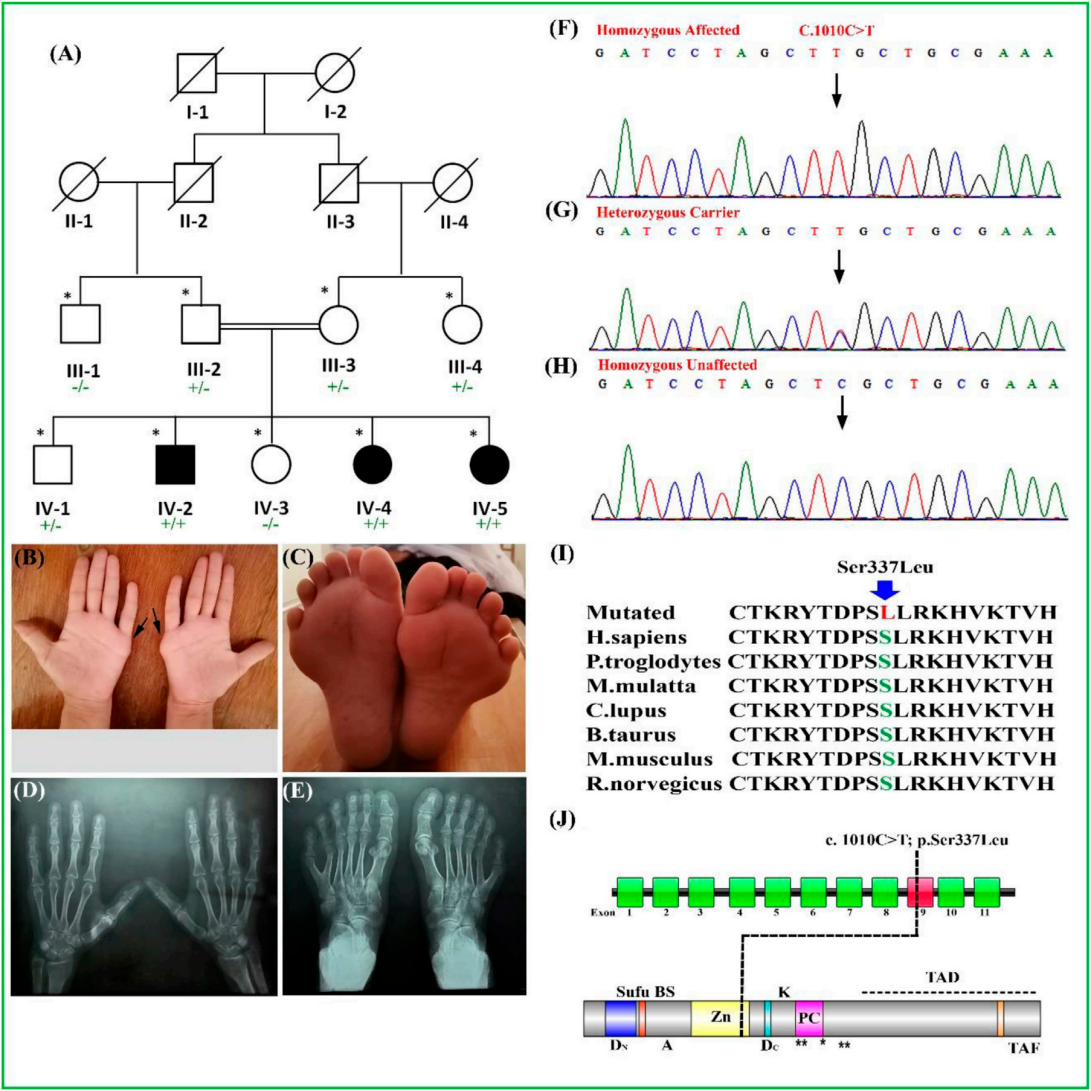
**(A)** Pedigree of a consanguineous family segregating PAP type A (PAPA) depicting an autosomal recessive manner. Filled and unfilled symbols represent affected and unaffected individuals, respectively. Double lines indicate a consanguineous union. A cross line on the symbol indicates a deceased individual. **(B–E)** Palmar view of hands of the affected individual. The extra digits were removed surgically. **(C)** Feet of the affected individual having PAPA. (D, E) Radiographs of hand and feet of the affected individual. **(F–H)** Sanger sequencing electropherograms of homozygous affected and heterozygous carriers followed by homozygous unaffected. The results showed segregation of the variant [*GLI1*_c.1010C > T; p(Ser337Leu)]) with the disease within family. **(I)** Partial amino acid sequence of GLI1 protein showing conservation of S337 amino acid across several species. **(J)** Schematic representation of the *GLI1* gene exons, with the position of variant (c.1010C > T) identified in the present study. Schematic representation of GLI1 protein and its domain reflecting position of identified variant. ZD, zinc-finger domains; AD, activator domain; D_N_, N-terminal degron; D_C_, C-terminal degron; Zn, zinc finger DNA binding domain; Sufu-BS, Sufu-binding site; PC, phosphorylation cluster; TAF, TAF-binding acidic activation motif; TAD, transcription activation motif; A, sumoylation site; K, acetylation site; PAP, post-axial polydactyly. ** Protein kinase A site.

GLI1 protein consists of 1,106 amino acids and acts as a mediator of hedgehog signaling pathway. GLI1 protein becomes active after proper binding of hedgehog molecule to its receptor, which ultimately results in transcription and further translation of target genes, which are associated with the limb development and patterning of digits (Ullah et al., 2019(b); Umair et al., 2017). GLI1 protein comprises of SUFU binding domain (consists of 111–125 amino acids), nuclear localization signal (consists of 380–420 amino acids), transactivation domain (consists of 1,020–1,091 amino acids), and a zinc finger domain (consists of 235–387 amino acids) (Figure 1J). The variant (p.Ser337Leu) identified in the present study is located in the highly conserved Zn finger domain, a DNA binding domain of GLI1 protein.

Our study recruited a consanguineous family segregating PAP type A in an autosomal recessive manner. Our finding revealed a novel homozygous missense variant by whole-exome sequencing (WES) followed by Sanger sequencing in *GLI1* gene. In addition to this, mutant modeling of this DNA binding protein was performed, which revealed a decline in the DNA binding activity of the mutant protein that ultimately affects the functional property of GLI1 protein.

## Materials and Methods

### Approval for Research and Ethical Consent

The institutional review board (IRB) of King Abdullah International Medical Research Center (KAIMRC), Riyadh, Saudi Arabia, approved this study. Written informed consent/approval forms were signed by elderly family members for conducting the research experiments and thereafter publication of all clinical data/results in the journals and conferences including radiographs and photographs of affected patients recruited in this study.

### Study Subjects and Family History

In our present study, a single family was recruited in which three members of the fourth generation (IV-2, IV-4, and IV-5) were exhibiting nonsyndromic PAP type A. A pedigree was drawn after communication with the most experienced and older family members (Figure 1A). The detailed family history and pedigree analysis depict the inheritance pattern of this disease in an autosomal recessive manner. Furthermore, clinical examination of all the affected family members mentioned above was performed in the local government hospital (Figures 1B–E).

### Clinical Description

The affected individuals were clinically evaluated in a local government hospital. All the affected individuals had classical PAP type A phenotypes. The age of the affected individuals (IV-2, IV-4, and IV-5) at the time of examination was between 25 and 35 years. All the affected individuals were born normally at full-term pregnancies without any additional complications. The affected individual (IV-4) showed bilateral PAP in both hands and feet (Figures 1B,C). Radiographic analysis showed that the origin of the extra digit came from the fifth metacarpal in both hands, while in the feet, the extra toes developed from the broad metatarsal (Figures 1D,E). The individuals (IV-2, IV-4, and IV-5) had the extra digits in their hands removed surgically. Partial webbing or syndactyly of the toes was observed in all the affected individuals (Figure 1C). All the affected individuals had normal weight, height, head circumference, sweating, vision, teeth, hearing, development, and body mass index (BMI). The laboratory blood tests such as sugar level, serum creatinine, urine protein test, and blood urea nitrogen were unremarkable. Facial features were unremarkable, the heterozygous carriers had normal hand/feet, and no other deformity was observed. Detail clinical description comparison is presented in Table 2.

**TABLE 2:** Clinical description of PAPA8-associated phenotypes as compared with the patients presented in the present study.

Study	Phenotype
Palencia-Campos et al. (2017)	*10 patients from 3 unrelated families
	*Family 1: (Turkish) Having bilateral PAP of hands and feet. The patient also had short stature, shortening of the long bones of the lower extremities, mild nail dysplasia, and atrial septal defect
	Family 2: (Turkish) Having bilateral PAP in hands and feet, short stature, and genu valgum
	Family 3: (Pakistani) Having bilateral PAP of the feet and unilateral PAP in hands (left)
	All the families revealed homozygous variants in *GLI1* gene
Ullah et al., 2019a	Family: (Pakistani) Having isolated bilateral pre-axial polydactyly type A in hands only
	The family revealed a homozygous variant in *GLI1* gene
Palencia-Campos et al. (2020)	95 independent probands with nonsyndromic PAP were screened for *GLI1* gene and revealed that 11.57% of these subjects have single heterozygous pathogenic variants in *GLI1*
Yousaf et al. (2020)	Investigated a large family segregating dominant form of PAP and identified a novel heterozygous missense variant (c.1064C > A; p.(Thr355Asn)) in gene *GLI1*
Present study	Identified a novel homozygous missense variant (c.1010C > T; p.Ser337Leu) in *GLI1* gene in a large family having 3 affected individuals presenting isolated-recessive PAPA type A
Umair et al	

Note. PAPA, post-axial polydactyly type A.

### Collection of Blood and Extraction of Genomic DNA

Approximately 3–5 ml of the peripheral venous blood samples was collected from the affected individuals (viz., IV-2, IV-4, and IV-5) and unaffected individuals (III-2, III-3, IV-1, and IV-3) in a cell-free DNA blood collection EDTA tubes (Streck, Omaha, NE, United States ); and each tube was labeled with a unique medical record number (MRN) and accession number.

For each blood sample, 2 ml of the buffy coat was collected after spinning whole blood at 4,000 rpm for 3–4 min. From each buffy coat sample (2 ml), genomic DNA extraction experiments were performed by using QIAamp DNA Blood Midi Kit from Qiagen (Hilden, Germany), according to the manufacturer’s protocol. The quantification of extracted DNA was determined by Qubit™ dsDNA HS Assay Kit (Cat. No. Q32851), as densitometric methods are not specific for DNA.

### Whole-Exome Sequencing

DNA of a single affected individual (IV-4) was exome sequenced using the Ion S5™/Ion S5™ XL Sequencers or the Ion Proton™ Sequencer (Thermo Fisher Scientific, Waltham, MA, United States ), and libraries were prepared using the Ion AmpliSeq™ Exome RDY Kits (Cat. No. A38262). WES was performed in the following steps, as per manufacturer’s protocol.

### Amplification of the Target DNA

For amplification, 56 µl of gDNA (100 ng) was taken and mixed with 14 µl of 5× Ion AmpliSeq™ HiFi Mix, and a total of 70 µl was made by adding nuclease-free water (NFW). Thereafter, the master mix was dispensed in a single row (12 Column) in the 96-well plate. The plate was transferred to the thermal cycler, and the cycling condition was 99°C for 2 min (1 cycle) followed by 99°C for 15 s and 60°C for 16 min (10 cycles).

### Partial Digestion of Amplicons

After completion of the amplification reaction, the 12 targets were combined into a single well no. 6, and thereafter, 6 µl of FuPa reagent was added to the combined target amplification reaction to bring the total volume up to 66 µl. Finally, the plate was transferred to the thermal cycler, and the program was set as 50°C, 55°C, and 60°C each for 20 min.

### Adaptor Ligation and Purification of Amplicons

After completion of partial digestion, 6 µl of diluted Ion Xpress™ Barcode Adapters was mixed, then 12 µl of switch solution was added, and finally at last 6 µl of DNA ligase was added. Finally, after completion of adaptor ligation, the library was purified by using 80 µl of AMPure™ XP reagent and 150 µl of freshly prepared 70% ethanol as per company protocol (Nakano et al., 2015).

### Quantification Loading of DNA Library Ion Chef™ Equipment

The quantification of the library was carried out by using the Ion AmpliSeq™ library by qPCR with the Ion Library TaqMan^®^ Quantitation Kit (Cat. No. 4468802) as per instruction given in the manufacturer’s protocol. The library was diluted to make the final concentration to ∼100 pM. The Ion Chef™ reagent was kept outside at room temperature for 45 min before loading of libraries. The other consumables and solution cartridge were placed at an appropriate position into the Ion Chef™ equipment (Thermo Fisher Scientific, United States , Cat. No. 4484177) (Palencia-Campos et al., 2020). Finally, 25 µl (∼100 pM) of the DNA library was transferred to position A of the reagent cartridge (Zhang et al., 2017).

### Initialization and Sequencing Run Into Ion S5™

The Ion S5™ ExT Sequencing Reagents cartridge was kept for 45 min to equilibrate the reagent at room temperature. After that, Sequencing Reagents cartridge, Ion S5™ ExT Wash Solution bottle, and Ion S5™ Cleaning Solution bottle were kept at an appropriate position in the Ion S5™ instrument (Thermo Fisher Scientific, United States , Cat. No. A27212). Finally, Ion 540™ chip (Cat. No. A34292) was used to perform initialization. After the completion of initialization, the chip was immediately transferred to the Ion S5™ instrument, and a sequencing run was performed (Zhang et al., 2017).

### Filtration of Variant Data Analysis

After completion of the sequencing run, the data were generated into a BAM file, which is automatically converted into a Variant Calling File (VCF) by the instrument, and the VCF was uploaded into a Base Space Illumina hub (https://basespace.illumina.com). Variant filtration steps also focused on the autosomal recessive mode of inheritance given priority as per the pedigree analysis. In addition to that, homozygous and heterozygous variants as well as pathogenic to likely pathogenic variants were also given priority during filtration steps.

Furthermore, different online software such as Varsome (https://varsome.com/), MutationTaster (http://www.mutationtaster.org/), FATHMM (http://fathmm.biocompute.org.uk/), and DANN (https://cbcl.ics.uci.edu/) were used to determine the pathogenicity of the identified variant in our study. The frequency of the identified variant in the general population was calculated by using available online databases including Exome Variant Server (EVS), 1,000 Genomes, ExAC, gnomAD, and/or in-house 2000 exome database.

### Segregation by Sanger Sequencing

To segregate the variants in affected and unaffected family members, Sanger sequencing was performed by using genetic analyzer 3500XL (Applied Biosystems, Foster City, CA, United States ) using standard methods (Umair et al., 2017).

### Mutant Modeling and DNA–Protein Interaction

The three-dimensional crystal structure of five-finger GLI1, a DNA binding protein in complex with DNA, was retrieved from Protein Data Bank (PDB) (https://www.rcsb.org/) with PDB Id, 2GLI. The S247L (Ser to Leu mutation at 247 position) mutant was generated through Pymol v.4.6.0^1^. DNA–protein interaction plots were generated through Nucplot as accessed on May 2021 using PDBsum (Luscombe et al., 1997; Laskowski et al., 2018). The illustrations of protein–DNA complex were prepared using Pymol v.4.6.0.

## Results

### Genetic Assessment

WES was performed for a single affected individual (IV-4), as described previously (Ullah et al., 2019(b)). The outcomes of WES data analysis and the filtration steps resulted in the identification of a novel homozygous missense mutation [c.1010C > T, p(Ser337Leu)] located on chromosome 12q13.3 (GRCh37) in exon nine of *GLI1* gene (Figure 1J). Until now, almost six different variants have already been reported in the *GLI1* gene causing PAP type A (PAPA) type 8 (PAPA8) as listed in Human Gene Mutation database (HGMD) (Table 4).

### Sanger Sequencing and Pathogenicity Validation

The bi-directional Sanger sequencing experiments were performed for affected and unaffected individuals by using exon specific primers (forward: 5′-CCT​GGA​TTT​ATG​GGA​CCT​CA-3′) and (reverse: 5′-GAA​TGG​ATG​GGT​TGG​GAA​GTA-3′), which showed correct segregation of a novel homozygous missense mutation (c.1010C > T) in the affected individuals, viz., IV-2, IV-4, and IV-5; heterozygous carrier in the individuals, viz., III-2 and III-3; and homozygous wild type in the individuals, viz., IV-1 and IV-3 (Figures 1F–H).

### Pathogenicity Index of the Novel *gli1* Variant

Pathogenicity of the novel missense variant (c.1010C > A; p. Ser355Leu) and its deleterious effects on the structure and function of protein was confirmed by using several available online software (Table 3).

**TABLE 3:** Depicting pathogenicity of the identified homozygous mutant (c.1010C > T; p.Ser337Leu) by using different online software.

SNO.	Prediction	Tool used	Prediction score
1	Disease causing	Mutation Taster	1
2	Damaging	SIFT4G	0
3	Damaging	PrimateAI	0.9066
4	Damaging	SIFT	0
5	Damaging	PROVEAN	−5.56, −5.37
6	Damaging	DEOGEN2	0.8772
7	Damaging	FATHMM-MKL	0.9464
8	Damaging	FATHMM-XF	0.953
9	Deleterious	LRT	0.0003499

Furthermore, the identified *GLI1* variant was screened in different online databases such as gnomAD (http://gnomad.broadinstitute.org/), ExAC (http://exac.broadinstitute.org), dbSNP and the 1,000 Genomes Project (http://www.internationalgenome.org/), or in-house 2000 exome database (https://www.ncbi.nlm.nih.gov/projects/SNP/). The identified novel variant was not detected in the homozygous state as mentioned in online tools. Until now, almost six different variants have already been reported for *GLI1* gene for PAPA8 as listed in HGMD (Table 4).

**TABLE 4:** List of mutations/variants already reported until now in *GLI1* gene for PAPA.

cDNA change	Protein change	Mutation type	AF(gnomAD)	Homozygous/heterozygous	Pathogenicity	References
c.337C > T	(p.Arg113Ter)	Nonsense	0.00000398	Homozygous	Pathogenic	Palencia-Campos et al. (2017)
c.1930C > T	(p.Gln644Ter)	Nonsense		Homozygous	Pathogenic	Palencia-Campos et al. (2017)
c.2340G > A	(p.Trp780Ter)	Nonsense		Homozygous	Pathogenic	Palencia-Campos et al. (2017)
c.1517 T > A	(p.Leu506Gln)	Missense	0.000211	Homozygous	Likely pathogenic	Ullah et al., 2019b
c.816G > T	p.(Trp272Cys)	Missense	0.00002125	Heterozygous	Deleterious	Palencia-Campos et al. (2020)
c.847 A > T	p.(Lys283*)	Nonsense	0.0	Heterozygous	—	Palencia-Campos et al. (2020)
c.883C > T	p.(His295Tyr)	Missense	0.0	Heterozygous	Deleterious	Palencia-Campos et al. (2020)
c.934 T > C	p.(Tyr312His)	Missense	0.000003985	Heterozygous	Disease causing	Palencia-Campos et al. (2020)
c.946G > A	p.(Glu316Lys)	Missense	0.000003983	Heterozygous	Disease causing	Palencia-Campos et al. (2020)
c.1139G > A	p.(Arg380Gln)	Missense	0.000003979	Heterozygous	Deleterious/disease causing	Palencia-Campos et al. (2020)
c.1064C > A	p.(Thr355Asn)	Missense	0.0	Heterozygous	Disease causing	Yousaf et al. (2020)
c.1010C > T	(p.Ser337Leu)	Missense	0.00000398	Homozygous	Deleterious/disease causing	Our finding

Note. PAPA, post-axial polydactyly type A.

### Mutant Modeling and DNA–Protein Interaction Analysis

**FIGURE 2:**
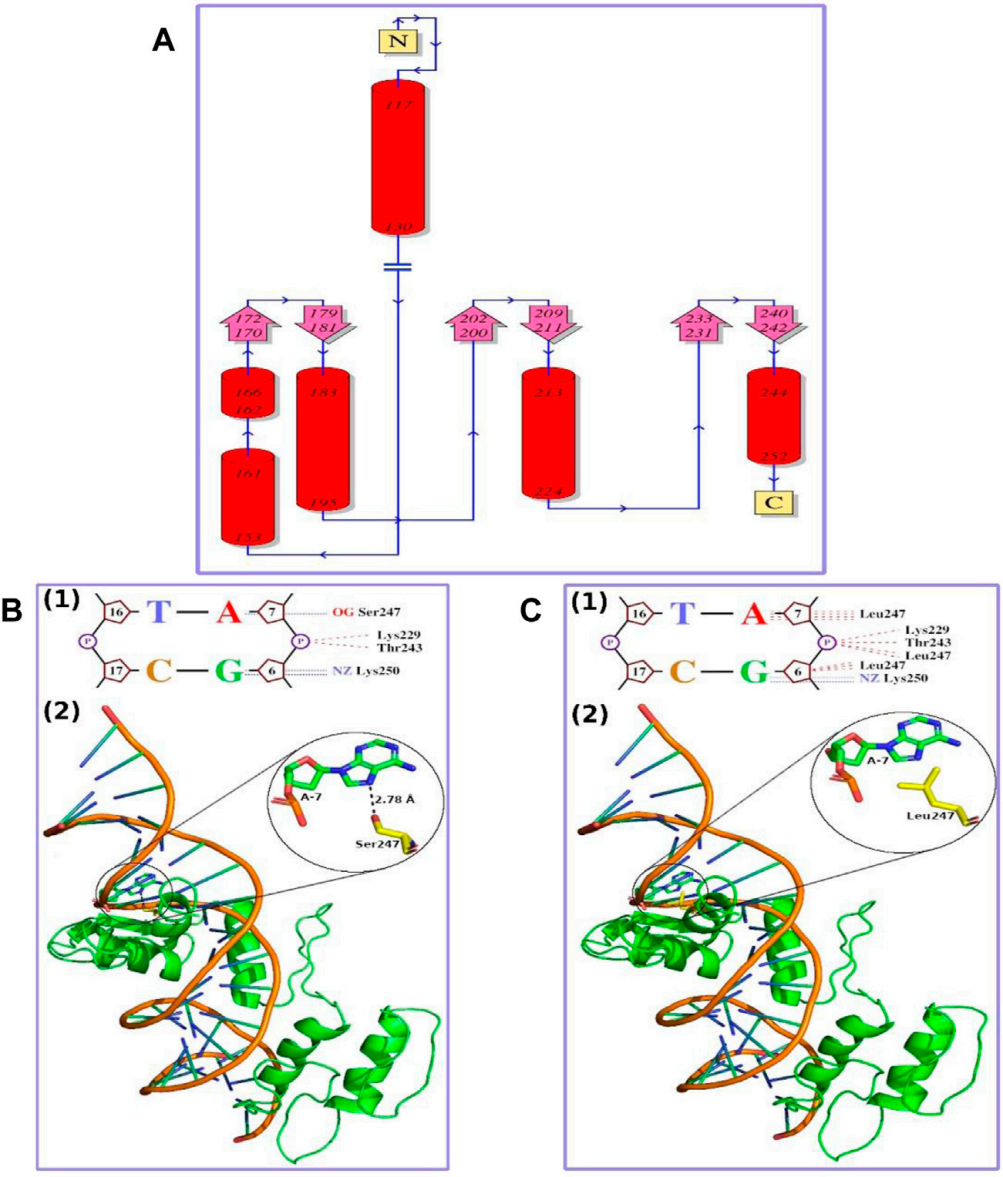
**(A)** Topology diagram of GLI1 protein. N and C show N- and C-terminal of the protein, respectively. Red cylinders marked with initial and final amino acid positions show alpha helices. Pink arrows show beta strands, and blue lines marked with direction (N-terminal to C-terminal) show loops or random coils. The mutation observed in this study (S247L) falls in C-terminal helix **(B)** Wild-type GLI1 protein in complex with native DNA. **(1)** Small part of DNA–protein interaction plot showing a hydrogen bond as blue dashed lines and non-bonding interactions as red dashed lines. “A,” “T,” “G,” and “C” represent nitrogenous bases; five-membered ring structure represents sugar; and “P” in circle represents phosphate group. Ser247 makes a hydrogen bond with nitrogenous base Adenine, A-7. **(2)** Ribbon representation of the protein and DNA complex. GLI1 protein is shown in green color, DNA phosphate backbone in orange color, and nitrogenous bases like rods in green color with blue ends. Ser247 residue in yellow forming a hydrogen bond with Adenine, A-7, in green is shown in sticks with heteroatoms colored differently (O-atoms, red; and N-atoms, blue). Close-up of the hydrogen bond interaction is provided in the inset **(C) **The S247L mutant GLI1 protein in complex with native DNA. **(1)** Small part of DNA–protein interaction plot showing a hydrogen bond as blue dashed lines and non-bonding interactions as red dashed lines. The “A,” “T,” “G,” and “C” represent nitrogenous bases; five-membered ring structure represents sugar; and “P” in circle represents phosphate group. **(2)** Ribbon representation of the protein and DNA complex. GLI1 protein is shown in green color, DNA phosphate backbone in orange color, and nitrogenous bases like rods in green color with blue ends. Leu247 residue in yellow forming non-bonding interaction with Adenine, A-7, in green is shown in sticks with heteroatoms colored differently (O-atoms, red; and N-atoms, blue). Close-up of Leu247 and Adenine A-7 is provided in the inset.

The topology of GLI1 protein is shown in Figure 2A. The mutational position (247 amino acid position) was falling in the C-terminal helix, and the mutation of Ser to Leu at this position was well tolerated in terms of structure, as Leu was not a helix breaker amino acid. We, therefore, considered the mutant structure similar to that of the wild type with the bound native DNA in the same conformation. The complete DNA–protein interaction plots for the wild type and mutant are provided as Supplementary File S1 and Supplementary File S2, respectively. The part of the DNA–protein interaction plots showing the 247th amino acid in the wild type and mutant are presented as Figures 2B1, 2C1, respectively. In wild-type GLI1 protein, Ser247 was forming a hydrogen bond with nitrogenous base Adenine-7 (A-7) with bond length of 2.78 Å (Figure 2B2). On the other hand, in mutant, Leu247 was interacting through non-bonding interaction with the nitrogenous base Adenine (A-7) (Figures 2C1–2), and additionally, it also formed non-bonding interactions with the sugar and the phosphate group of the previous nucleotide Guanine (G-6) (Figure 2C1). The hydrogen bond is one of the strongest intermolecular attractions and is much stronger than the non-bonding interactions (Perrin and Nielson, 1997). Further, this hydrogen bond (Ser O−H·N Adenine) was the strongest one (strongest among “O−H···N, O−H···O, N−H···N, and N−H···O” found in the living system), and the bond length was also small (2.78 Å). Therefore, the loss of this hydrogen bond due to mutation might result in weak interaction between DNA and the protein and may lead to alteration in the activity of the protein.

## Discussion

PAP is a congenital malformation of limb development characterized by an extra finger or duplication of the fifth digit of the autopod. This condition could occur as an isolated entity or a part of a complex syndrome. Sonic hedgehog (Shh) controls the number of digits of the autopod during early embryonic development by regulating the activity of GLI1, GLI2, and GLI3 (Tickle and Towers, 2017). GLI proteins bind DNA using five consecutive C2H2 zinc finger (ZF) motifs, and GLI1 is exclusively involved in transcriptional activation. Since GLI1 transcript levels increase in response to Hh ligands, it serves as a target and Hedgehog pathway amplifier (Lee et al., 1997). Thus, ultimately, the balance between GLI repressors and activators determines the outcome of Hh signaling in each cell (Ruiz et al., 2007).

GLI1 (GLI Family Zinc Finger 1) is a member of the Kruppel family of zinc finger proteins. Variants in *GLI1* gene have been associated with polydactyly PAPA8 and PPD type I (Ullah et al., 2019(a); Palencia-Campos et al., 2020). In the present investigation, using WES followed by bi-directional Sanger sequencing, we have identified a novel biallelic missense variant (c.1010C > T; p(Ser337Leu)) in exon nine of *GLI1* gene (NM_001167609.1) located on chromosome 12q13.3 in a consanguineous Pakistani family exhibiting hallmark features of PAPA. The variant (p(Ser337Leu)]) is considered disease causing by several online bioinformatics tools (Table 3) and is highly conserved across multiple species (Figure 1I). However, the above novel variant was not detected in the homozygous state in different online public databases such as gnomAD, ExAC, dbSNP, 1,000 Genomes Project, or in-house 2000 + exome database.

The highly conserved polar Serine amino acid at position 337 is substituted by a non-polar aliphatic leucine amino acid. This substitution, signposted by the 3D structural analysis, might decline the DNA binding. Thus, alteration in the protein’s function might lead to abnormal digit features in the affected individuals (Figures 2B,C).

In the present investigation, bilateral PAP in both hands and feet with complete development of extra digit was observed in all affected members of the family (Figure 1B). Palencia-Campos et al. reported three homozygous disease-causing variants in three families segregating PAPA with overlapping EVC syndrome phenotypes (Ullah et al., 2019(a); Palencia-Campos et al., 2020). Ullah I. et al. (2019) reported a Pakistani family segregating PPD in recessive fashion and having a missense variant (c.1517 T > A; p. Leu506Gln) in *GLI1* gene. PPD features were not observed in patients reported here. Isolated PAPA features observed in the present family were a mirror to the features reported previously [Umair et al., 2017 (Ullah et al., 2019(a); Umair, Wasif et al., 2019; Hayat et al., 2020, Umair et al., 2021]. Features such as PAPA restricted to the lower limbs (Umair et al., 2017) and overlapping EVC features (Palencia-Campos et al., 2020) reported previously were not observed in our patients. Detailed clinical comparisons of all the families reported having GLI1 variant associated with PAP is presented in Table 2.

Recently, heterozygous variants in *GLI1* gene have been associated with isolated PAP A/B (Palencia-Campos et al., 2017). It is highly likely that the phenotypes produced by the GLI1 heterozygous variants in several families resulted from clinical heterogeneity, incomplete penetrance, variable expressivity, and role-played by the modifier genes.

The present study has few limitations, including a small sample size available for genetic testing in familial cases; however, Sanger sequencing of the nine family members from the same family fulfilled this limitation. Second, most affected individuals surgically removed the extra digit, so images were unavailable for clinical evaluations. Third, functional and expression studies were not performed. Still, this study might help in genotype–phenotype correlations and help us understand the pathophysiology underlying the GLI1 pathogenesis.

In conclusion, we detected a novel missense variant in *GLI1* gene resulting in an isolated recessive PAPA8. Despite the inbreeding population, we found no evidence for another disease-causing variant in the affected individual’s exome data, thus decreasing the probability of a dual molecular diagnosis (digenetic). Our data further support the evidence that biallelic variants in GLI1 cause recessive PAP in humans. Identifying variants in recently known polydactyly genes provides clinicians, genetic counselors, and researchers valuable clinical information. These might help in genetic counseling of the affected family and prevent such disorders in upcoming generations.

## Data Availability

The data presented in the study are deposited in the LOVD: (https://www.lovd.nl/2.0/index.php) repository, Individual ID: 00384519, variant ID:0000812862.

## Footnotes

1
https://pymol.org/2/
