# Supplementary material for: A Novel Homozygous Missense Mutation in the Zinc Finger DNA Binding Domain of GLI1 Causes Recessive Post-Axial Polydactyly
Source: Front Genet. 2021 Oct 15;12:746949. doi: 10.3389/fgene.2021.746949 (PMC8554680; doi:10.3389/fgene.2021.746949)
Supplement: Supplementary file 1 [file Image2.pdf]

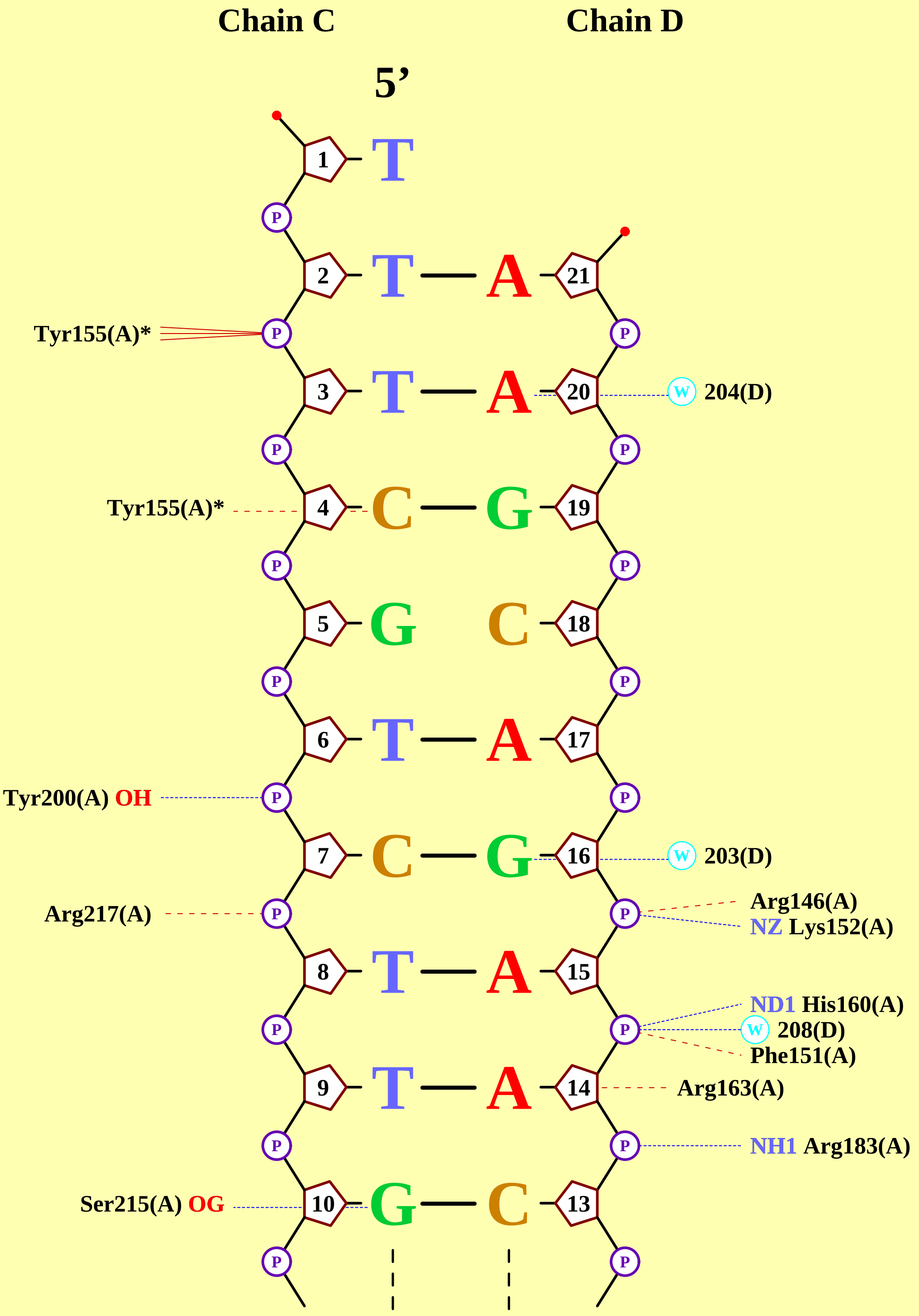

Key

3 Backbone sugar and base-number

P Phosphate group

* Residue/water on plot more than once

Hydrogen bond to DNA

Nonbonded contact to DNA (< 3.35A)

88 W Water molecule and number

file

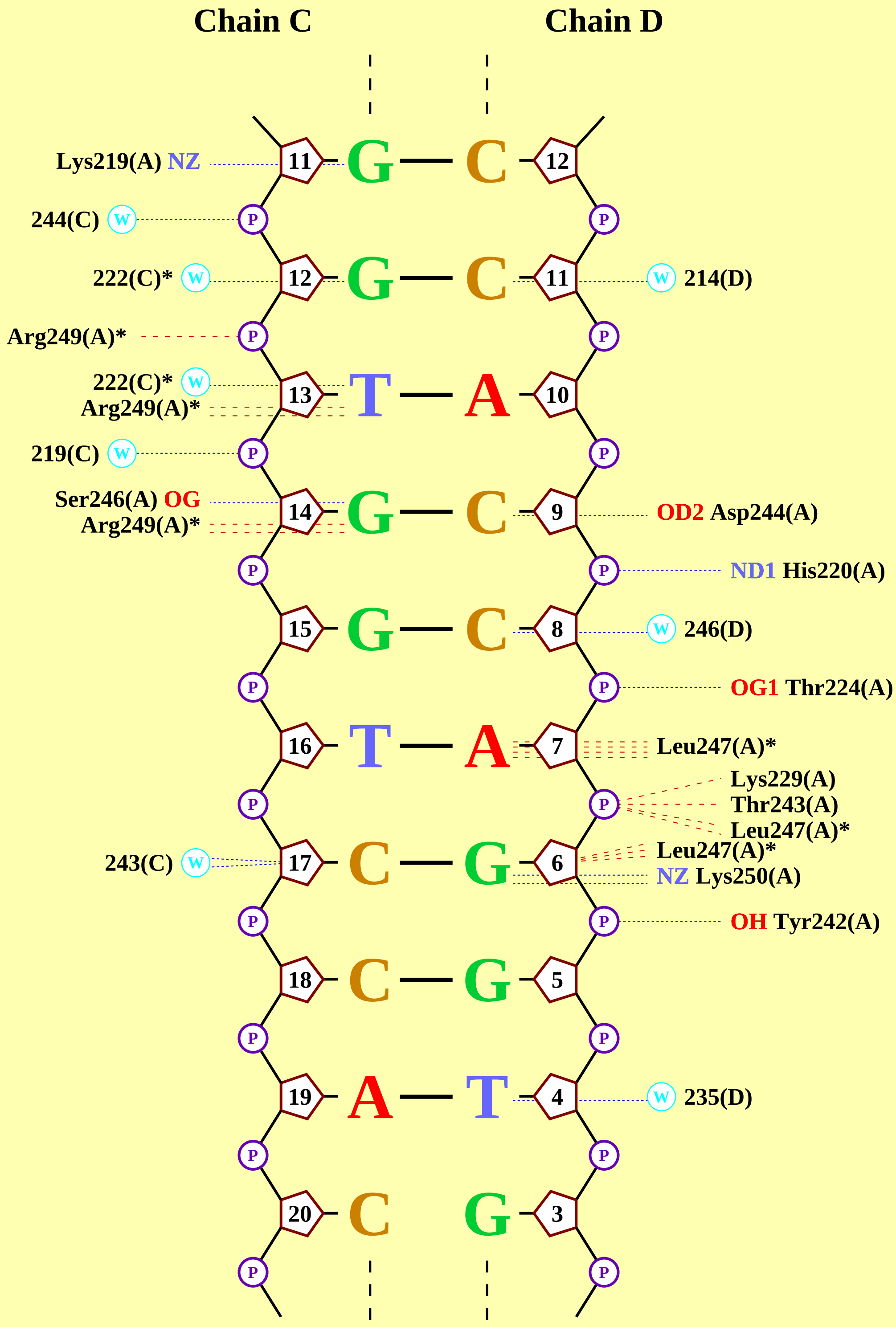

Key

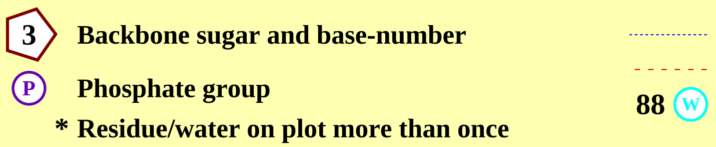

3 Backbone sugar and base-number

P Phosphate group

* Residue/water on plot more than once

Hydrogen bond to DNA

Nonbonded contact to DNA (< 3.35A)

88 W Water molecule and number

file

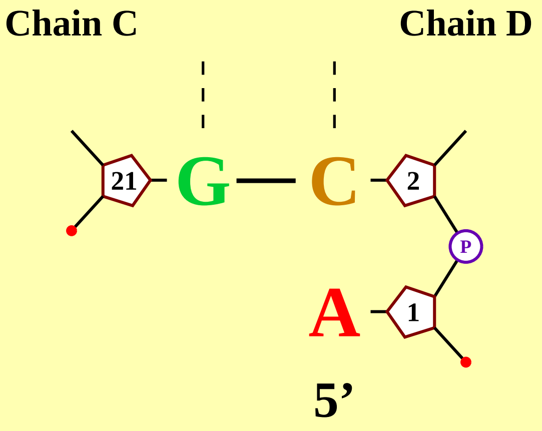

Key

Backbone sugar and base-number

Phosphate group

* Residue/water on plot more than once

Hydrogen bond to DNA

Nonbonded contact to DNA (< 3.35A)

88 Water molecule and number

file
